# Effect of (Poly)phenols on Lipid and Glucose Metabolisms in 3T3-L1 Adipocytes: an Integrated Analysis of Mechanistic Approaches

**DOI:** 10.1007/s13679-025-00656-6

**Published:** 2025-08-06

**Authors:** Marco Rendine, Mirko Marino, Daniela Martini, Patrizia Riso, Peter Møller, Cristian Del Bo’

**Affiliations:** 1 Division of Human Nutrition, Department of Food, Environmental and Nutritional Sciences (DeFENS), Università Degli Studi Di Milano, Milano, Italy; 2 Department of Public Health, University of Copenhagen, Copenhagen, Denmark

**Keywords:** 3T3-L1 adipocytes, (poly)phenols, Glucose uptake, Lipid metabolism, Thermogenesis

## Abstract

**Purpose of Review:**

This systematic review aims to elucidate the effects of (poly)phenols (PPs) on mature 3T3-L1 adipocytes via the regulation of lipid and glucose metabolism.

**Recent Findings:**

PPs can modulate glucose uptake, reduce intracellular lipid content and enhance lipolytic activity in mature 3T3-L1 adipocytes. These effects are mediated through changes at both gene expression level (e.g. *Ppara* and *Sirt1*) and protein level (e.g. activation of AMPK and adiponectin levels). However, there is no consensus on the concentrations at which PPs exert their anti-lipogenic activity, and it remains unclear whether different PPs activate distinct molecular pathways.

**Summary:**

PPs are a diverse group of plant-derived secondary metabolites with recognized anti-obesogenic potential. While their inhibitory effects on adipogenesis are well established, their role in modulating lipid metabolism in fully differentiated adipocytes remains less well understood. Emerging evidence from studies on mature 3T3-L1 adipocytes indicates that PPs can influence key metabolic processes, including lipid storage and mobilization. These findings highlight the potential of PPs as modulators of adipose tissue metabolism, while also emphasizing the need for translational research to clarify their mechanisms of action and therapeutic efficacy *in vivo*.

**Supplementary Information:**

The online version contains supplementary material available at 10.1007/s13679-025-00656-6.

## Introduction

(Poly)phenols (PPs) are a large group of natural compounds found in plants, serving as secondary metabolites with various biological activities. These compounds are prevalent in a wide range of foods and beverages, including fruits, vegetables, tea, and coffee, contributing to their health benefits. Growing evidence has demonstrated the potential of PPs in exerting antiadipogenic (inhibiting the formation of fat cells) and anti-lipogenic (reducing lipid accumulation) effects, which are crucial in the context of obesity and related cardio-metabolic disorders [1].

Most of the recent understanding of the anti-obesogenic effects of PPs derives from in vitro studies utilizing the murine 3T3-L1 cell line, a subclone of cells originally isolated and cultured from a mouse embryo (called 3T3 cells). Both 3T3 and 3T3-L1 clones display fibroblast-like characteristics. Notably, the 3T3-L1 subclone can differentiate into adipocytes when stimulated with hormonal inducers such as insulin, dexamethasone, and 3-isobutyl-1-methylxanthine. Consequently, it is considered to be a pre-adipocyte cell line and has been widely used as an in vitro model to study cellular mechanisms related to obesity [2].

However, it is important to distinguish between the biological differences between using PPs in differentiating 3T3-L1 cells during adipogenesis versus their application in fully differentiated adipocytes [3]. Adipogenesis is the process by which pre-adipocytes differentiate into mature fat cells. Numerous studies have documented that PPs can effectively reduce the differentiation of 3T3-L1 cells into adipocytes, thus preventing the formation of new fat cells. In contrast, the impact of PPs on fully differentiated (mature) adipocytes is less explored and understood. Mature adipocytes represent the final stage of adipogenesis, and they are the primary cells involved in lipid storage and metabolism. In adipocyte-based in vitro models, such as differentiated 3T3-L1 cells, lipid accumulation typically occurs as part of the differentiation process, leading to the formation of intracellular lipid droplets. During the differentiation, 3T3-L1 cells undergo changes to develop the ability for *de novo* lipogenesis, in which glucose is converted into fatty acids and subsequently esterified with glycerol to form triglycerides. The endogenous lipid synthesis is essential for lipid droplet formation and adipocyte maturation [4].

The modulation of lipid and glucose metabolism in adipocyte-based in vitro models is essential for understanding the anti-lipogenic potential of PPs. Furthermore, these anti-obesogenic effects are accompanied by thermogenesis and mitochondrial biogenesis, which are associated with increased energy expenditure [5]. Despite the promising anti-adipogenic properties of PPs, their efficacy in altering lipid metabolism in mature adipocytes, which directly relates to the reduction of lipid accumulation and improvement of metabolic health, remains to be fully elucidated. Moreover, there is significant variability in the concentrations of PPs used across different studies, leading to inconsistencies in the reported outcomes.

In human studies, physiological plasma concentrations of (poly)phenols and their metabolites typically range between 0.1 and 10 µM, depending on several factors including compound chemical structure, bioavailability, metabolism, and amount introduced through the diet [6]. For example, after the ingestion of PP-rich foods such as berries, cocoa, or tea, peak plasma concentrations of flavonoids and phenolic acids are usually observed within 1–2 h and the concentration is generally below 5 µM [6]. In contrast, many in vitro studies employ concentrations between 10 and 100 µM, which exceed the amount regularly achieved through the diet by reflecting a more pharmacological exposure [7]. This discrepancy underscores the importance of contextualizing in vitro findings with physiologically relevant concentrations to better assess translational potential.

This review aims to provide a deeper understanding of the anti-obesogenic potential of PPs and their applicability in obesity management as well as also highlight the current limitations and suggest directions for future research to harness the potential role of PPs in counteracting obesity and associated metabolic disorders. Given these gaps in knowledge, this systematic review aims to comprehensively evaluate the effects of PPs on mature 3T3-L1 adipocytes. Specifically, we seek to: (1) summarize the evidence on the impact of PPs on lipid and glucose metabolism in fully differentiated adipocytes; (2) identify the effective concentrations at which PPs exert their anti-lipogenic activities; (3) discuss the potential mechanisms through which PPs modulate lipid metabolism in mature adipocytes.

## Materials and Methods

This review was registered in the Open Science Framework (DOI 10.17605/OSF.IO/NV873). The review question was formulated according to the acronym PICOS (population, intervention, control, outcomes, and study design) (Table 1).

**Table 1:** PICOS strategy applied to the present systematic review

PICOS	Description
Population	Differentiated/mature 3T3-L1 adipocytes cultured in DMEM
Intervention	(Poly)phenols or (poly)phenols-rich extracts
Comparison	Untreated differentiated / mature 3T3-L1 adipocytes
Outcomes	Lipogenesis, adipogenesis, thermogenesis/mitochondrial biogenesis, and glucose metabolism
Types of studies included	In vitro (cell culture studies)
Research question	Can (poly)phenols exert an anti-obesogenic activity in differentiated / mature 3T3-L1 adipocytes as a model of obesity?

### Search Strategy

The search strategy was based on PRISMA guidelines for systematic reviews [8]. The literature review was conducted in three databases (PubMed, Scopus, and Web of Science (Web of Knowledge)). The first phase established an investigation according to the features and strategies of each electronic database (Supplementary File 1; S1). Two researchers first screened the titles and then the abstracts (MR and MM). To retrieve the publications the search strategy was based on selected keywords/ MeSH terms organized into three blocks: Block 1 (cell model): “3T3-L1 [MeSH terms]” OR “3T3-L1 adipocytes” AND Block 2 (treatments): “polyphenols” OR “phenolic compounds” OR “flavonoids”; AND Block 3 (outcomes): “lipogenesis” OR “adipogenesis” OR “lipid accumulation” OR “anti-obesity” OR “anti-obesogenic” OR “obesity”. We applied the topic strategy for PubMed and Web of Science (all fields), and Scopus (title/keyword/abstract) applying filter language (English). The references retrieved from the searches were organized in a Microsoft Excel folder.

### Study Selection

Duplicates were eliminated using the “remove duplicates” function in Microsoft Excel and then cross-checked manually. In the initial phase, two independent authors (MR and MM) screened articles based on their titles and abstracts, applying eligibility criteria outlined by the PICOS question. Discrepancies among the reviewers were resolved by consulting a third reviewer (CDB). Subsequently, the full texts of the selected studies were obtained and independently evaluated for eligibility by the same two authors (MR and MM). Any differences in judgment were settled through discussion among the reviewers and solved by an independent expert (CDB) as needed.

### Inclusion Criteria

Inclusion criteria were established through the PICOS strategy. Population (P): differentiated 3T3-L1, cultured in Dulbecco’s Modified Eagle Medium (DMEM). The use of this cell culture medium in the studies was identified as an eligibility criterion, according to the ATCC base medium recommendation for this cell line (American Type Culture Collection—ATCC, 2018) [9]. Intervention (I)/Exposure: Any treatment with (poly)phenols or (poly)phenols-rich extract. Comparator(s)/control (C): differentiated 3T3-L1 adipocytes not treated with (poly)phenols or (poly)phenols-rich extracts (control group). Outcome (O)/measure(s): Levels of lipid accumulation (Oil Red O staining or concentration of intracellular lipids), glucose uptake, glycerol released, or markers related to at least one of the following molecular pathways: adipogenesis/lipogenesis, glucose metabolism, and thermogenesis. Study designs (S) included: Only original articles from in vitro studies (differentiated 3T3-L1 adipocytes), with separate control cells. Articles written in English, published from January 1, 2000, to December 31, 2024.

### Exclusion Criteria

The exclusion criteria of the PICOS strategy were also applied, following the recommended sequence of PICOS. These criteria were selected based on the main guidelines for cell culture (American Type Culture Collection— ATCC, 2018) [9]. In the first phase of eligibility, the title and abstract were read, and the following exclusion criteria were applied: (1) studies not using the 3T3-L1 cell line (2) intervention not based on (poly)phenols or (poly)phenols-rich extracts; (3) studies not reporting “lipid accumulation or glycerol release or glucose uptake” or generally not referring to “lipogenesis or adipogenesis or glucose metabolism or thermogenesis”; (4) other inapplicable studies/publications, including animal models, clinical trials or other studies involving humans, reviews, Guidelines, Letters, Editorials, Comments, News, conference abstracts, thesis, and dissertations. In the second phase, the full text was read (unless unavailable), and additional exclusion criteria were introduced in addition to the previously established criteria: (5) use of co-culture system; (6) (poly)phenols administered before or during adipocyte differentiation; (7) timing of treatments not specified; (8) (poly)phenols administered alongside additional treatments (e.g. inflammatory or oxidative stimuli); (9) outcomes not of interest (10) full text unavailable.

### Data Extraction

Data were extracted by two independent authors (MR and MM) and inserted into a standardized table. The extracted data were validated by a third reviewer (CDB) who also resolved any discrepancies during the process. The main information collected is reported as Supplementary File 2 (S2) and includes: the 1st author and year of publication; Seeding conditions, including cell density and experimental plates; Cell culture conditions, including type cell culture media, glucose concentration, and any supplementation present in the culture media; cytotoxicity assay; treatments, including type of compounds/extracts tested, concentrations and day at which treatment was administered with respect to the beginning of adipocytes’ differentiation; Control, including the type of vehicle used; Duration of treatments; Assays; Main findings, including results reported in the original papers related to cellular phenotypes (lipid accumulation), findings regarding mechanisms of action (glucose uptake, lipolytic activity, and markers of molecular pathways), and the direction of the observed modulations (reduction or increase).

## Results and Discussion

**Fig. 1:**
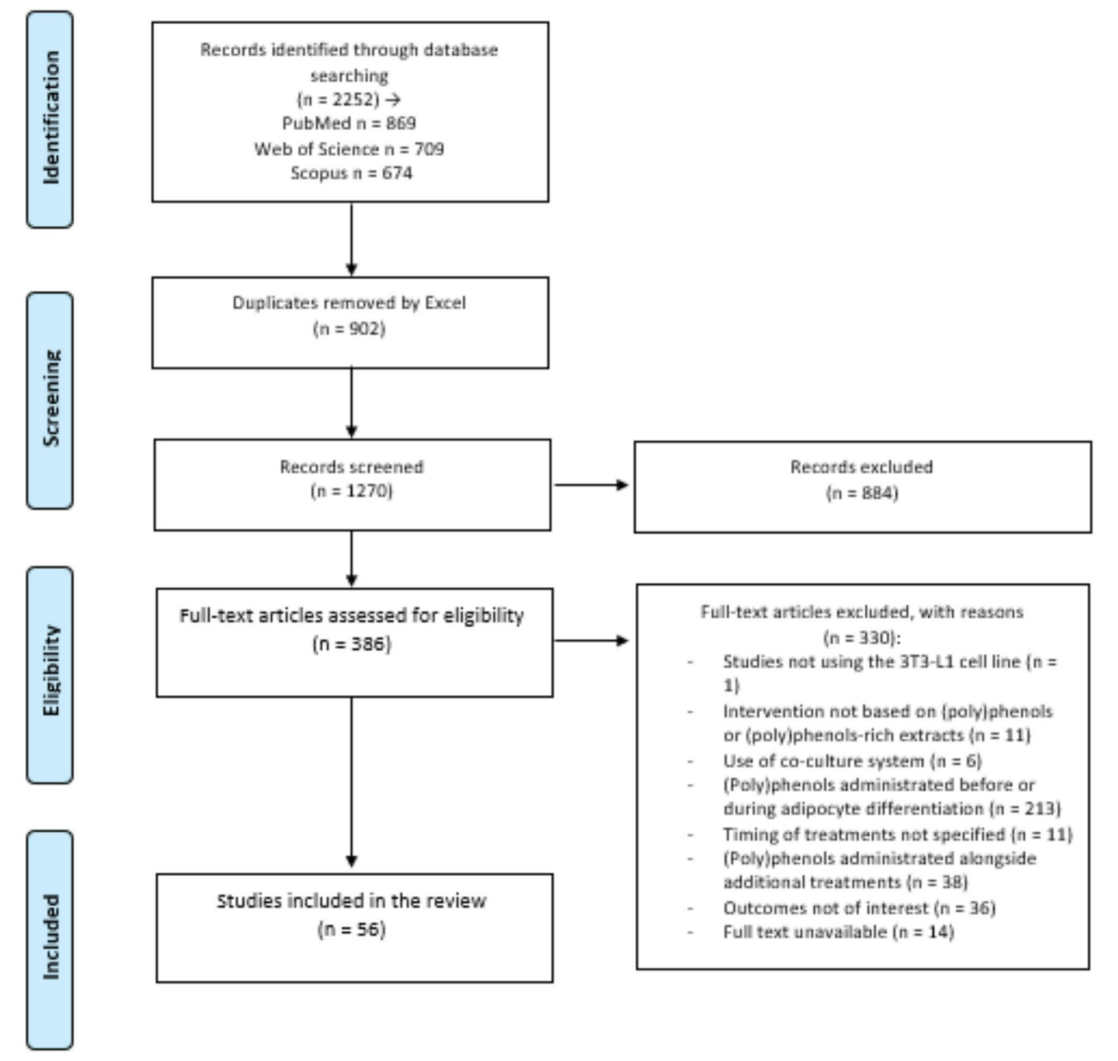
Prisma flow diagram of the systematic review

The research yielded 2,252 articles. This number was reduced after the application of an automatic function to remove duplicates (Microsoft Excel) and further checked manually leaving a total of 1,270 articles to be submitted to the first phase of screening by the application of eligibility criteria (Fig. 1). After applying the first round of exclusion criteria (title and abstract), a total of 884 articles were excluded, leaving 386 articles to be assessed by reading the full text. In the second round, 330 were excluded after full-text screening, as they did not meet the previously defined inclusion criteria. The remaining 56 articles were included in this review and sent for data extraction (S2).

### Characterization of (poly)phenols Tested among the Studies

The main characteristics of the studies are reported in Table 2. Thirty-one studies used single phenolic compounds, eighteen tested only PP-rich extracts (or PP-rich fractions), and seven studies used both extracts and single compounds.

The studies investigated the effects of various classes of (poly)phenols, including flavonols (*n* = 8) [10–17], flavanones (*n* = 6) [10, 13, 18–21], flavanonols (*n* = 2) [12, 22], flavones (*n* = 7) [12, 13, 23–27], flavan-3-ols (*n* = 6) [10, 28–32], isoflavones (*n* = 2) [21, 33], anthocyanins (*n* = 1) [34], stilbenes (*n* = 11) [13, 35–44], and phenolic acids (*n* = 3) [13, 45, 46].

The concentrations of the PPs tested in the different studies varied in the range of 0.001-600 µM, with some studies using different units of measurement (e.g. µg mL^− 1^ or ppm). Studies were divided based on the concentration of compounds into two categories: studies using physiological concentrations (≤ 2 µM), which can be found in human plasma after the consumption of dietary portions of PP-rich foods, and pharmacological concentrations (> 2 µM), which could theoretically be achieved through supplementation. Most of the studies used pharmacological concentrations of PPs to treat adipocytes. Specifically, only seven studies tested concentrations close to physiological ones or slightly above (≤ 2 µM).

Overall, the most frequently investigated PP classes were stilbenes, flavonols, flavones, flavanones, and flavan-3-ols. Among these, resveratrol (stilbene), quercetin (flavonol), and genistein (isoflavone) were the most tested individual compounds. These PPs are not only widely studied but are also abundant in common dietary sources, such as red grape (resveratrol, 0.16-24 mg/100 g), onions (quercetin, ~ 300 mg/100 g), and soy (genistein, 5.6 to 276 mg/100 g,) [66–68]. In addition, several studies investigated the effects of PP-rich extracts, including those derived from commonly consumed foods, such as berries. However, it is important to highlight that the use of extracts and/or fractions in cellular models such as adipocytes may raise concerns regarding biological plausibility. In fact, from a physiological point of view, complex food extracts undergo extensive digestion, absorption, metabolism, and biotransformation, particularly in intestinal and hepatic compartments, before reaching peripheral tissues. As a result, it is highly unlikely that raw extracts, as applied directly to cultured adipocytes, would come into contact with adipose tissue in vivo in their original form. This limits the translational relevance of such experiments in an in vivo context. Therefore, while these studies can provide valuable mechanistic insights, their results should be interpreted with caution in terms of dietary applicability. Highlighting these considerations, as well as the most frequently studied and diet-relevant PPs, may enhance the practical value and interpretability of the findings.

**Table 2:** Number of studies segregated into types of (poly)phenols, concentration range and main compounds tested within each class

Classes of (Poly)phenols	Number of Studies	Concentration Range	Main compound(s) or extract tested and relative concentration	Ref
Flavonols	8	0.1–600 µM	Quercetin and its metabolites (0.1–600 µM) Rutin (5-500 µM)	[10–17]
Flavanones	6	0.5–350 µM	Naringenin (10–350 µM)	[10, 13, 18–21]
Flavanonols	2	1–100 µM	Taxifolin (75–600 µM)	[12, 22]
Flavones	7	0.001–600 µM	Luteolin (10–600 µM)	[12, 13, 23–27]
Flavan-3-ols	6	0.2–150 µM	Epicatechin (0.5–10µM) Epigallocatechin gallate (0.1–10µM)	[10, 28–32]
Isoflavones	2	5–100 µM	Genistein (5-100 µM)	[21, 33]
Anthocyanins	1	5–20 µg mL^− 1^	Fraction (5–20 µg mL^− 1^)	[34]
Stilbenes	11	1–100 µM	Resveratrol (1-100 µM)	[13, 35–44]
Phenolic acids	3	0.5–100 µM	Protocatechuic acid (0.5-5µM	[13, 45, 47]
Extracts	25	0.001–1000 mg mL^− 1^	Grape seed procyanidins extract (140–210 mg/L)	[10, 12, 22, 32, 34, 46–65]

### Modulation of Lipid and Glucose Metabolisms

The following sections summarize the effects of extracts and compounds on lipid storage and glucose uptake and identify the main mechanisms of action by which different classes of (poly)phenols affect lipid and glucose metabolism (S2).

#### Effects of (poly)phenols on Adipocytes’ Lipids Storage

Adipocytes play a crucial role in energy storage by accumulating triacylglycerols (TAGs) in cytosolic lipid droplets. The TAGs are mobilized and broken down into fatty acids and glycerol when the body requires energy. Lipid storage is evaluated in cell culture, particularly in adipose cells, primarily through staining techniques targeting cellular lipids (e.g., Oil Red O assay) and by assays that involve the isolation and quantification of intracellular TAGs.

In this review, thirty-one studies investigated the effects of PPs on intracellular lipid content (lipid accumulation and/or TG content) [11–14, 16, 18, 22, 23, 25, 28–30, 34, 36, 37, 39, 41–44, 47, 49, 50, 52–58, 65]. Only three papers reported that (poly)phenols were unable to affect intracellular lipids [11, 53, 65] and one study showed an increase in lipids after treatment with PP extracts [28]. The remaining papers evidenced a reduction of intracellular lipid content after treatment with PPs. The reduction of cellular lipids was evidenced in eleven papers after treatment with PP-rich extracts, while nineteen studies evidenced an effect on lipids exerted by single phenolic compounds. Cellular lipid reduction was consistent across different classes of (poly)phenols, including flavonols (e.g., myricetin, rutin, kaempferol, quercetin and metabolites) [11–14], flavones (e.g., luteolin, isoorientin, apigenin) [12, 13, 23, 25], anthocyanins [34], flavanones (e.g., naringin, hesperidin) [13, 18], flavanonols (e.g., ampelopsin, taxifolin) [12, 22], phenolic acids (e.g., p-coumaric acid, ellagic acid, ferulic acid, gallic acid, vanillic acid, chlorogenic acid) [13], and stilbenes (e.g., piceatannol, resveratrol) [13, 36, 37, 39, 41–44]. However, there is not sufficient experimental evidence to conclude that certain PPs are particularly effective in reducing lipid content in mature adipocytes. From a physiological perspective, PP-extracts are unlikely to reach adipocytes in their native form; therefore, studies investigating the effects of individual compounds are more relevant in terms of mechanistic plausibility. Finally, it should be noted that most of the available findings, particularly those involving single compounds, have been obtained using supraphysiological or pharmacological concentrations.

#### Effects of (poly)phenols on Lipolysis

Lipolysis is a crucial metabolic process involving the hydrolysis of triglycerides into non-esterified fatty acids (NEFA) and glycerol [69]. Catecholamines, including norepinephrine (NE) and epinephrine, are the main physiological stimulators of lipolysis, while other hormones can also affect lipolytic activity. Several studies have investigated the effects of extracts and single compounds on the extent of lipolysis in mature 3T3-L1 adipocytes, evaluating the spontaneous and/or NE-induced activation of the lipolytic pathway. In cell culture systems, lipolytic activity is proportionally related to the release into cell supernatants of the two products derived by the lipolytic pathway: NEFA and glycerol. In the studies included in this review, adipocytes were not lipid-loaded with exogenous fatty acids, instead, lipid accumulation was achieved through glucose metabolism via *de novo* lipogenesis (DNL).

This review includes six studies that evaluated the effect of different PP-rich extracts on lipolysis. Among these, one showed no effects [51], four papers reported an increase of both spontaneous [32, 59, 62] and NE-induced glycerol release [65], and one study reported a decrease of both spontaneous and NE-induced lipolysis [60].

Regarding the evaluation of the impact of single phenolic compounds on lipolytic activity, eleven studies evaluated the effects of various (poly)phenols [16, 20, 21, 23, 25, 29–32, 40, 46]. Mainly, studies focused on the effect of flavan-3-ols on lipolysis. In three studies, epigallocatechin-3-gallate was shown to increase lipolysis [30, 31, 46]. Additionally, an increase in lipolysis was observed following treatment with rutin polymers and xanthohumol [16, 20]. Similarly, green tea catechins and the isoflavone genistein were also shown to enhance lipolysis, as evidenced by an increase in norepinephrine-induced release of NEFAs and/or glycerol from adipose cells [21, 40]. Ko et al. [29] reported an increase in the spontaneous release of glycerol, but not of NEFA, after treatment with theaflavin-3,3′-digallate. Finally, a single study testing catechin and epicatechin reported that compounds did not affect lipolysis [32].

Additionally, other PP classes have been investigated for their effect on lipolysis, using glycerol release or 3’,5’-cyclic adenosine monophosphate (cAMP) levels as indicators of biological effect. The cAMP is generated as a second messenger within adipocytes upon beta-adrenergic receptor-stimulation on the cell membrane and it activates hormone-sensitive lipases within the cell. Treatment with flavones (i.e., sudachitin, nobiletin, isoorientin, xanthohumol) and phenolic acids (i.e., chlorogenic acid) enhanced the glycerol release and/or cAMP levels, indicating an increased adipocytes’ lipolytic activity [16, 19, 21, 42]. In addition, supplementation with isoflavones (i.e. genistein) increased both the spontaneous and NE-induced glycerol release [21].

#### Effects of (poly)phenols on Insulin-Dependent and Insulin-Independent Glucose Uptake

Glucose is one of the primary energetic sources in vivo and essential for the maintenance of cell growth in vitro. Unsurprisingly, glucose supplementation affects lipid accumulation in cultured adipose cells. The glucose uptake is enhanced by stimulation with insulin, facilitated by glucose transporter member 4 (GLUT4) in adipocytes. However, there is also an insulin-independent glucose uptake in adipocytes. This occurs primarily through glucose transporters including GLUT1, which are constitutively expressed and mediate basal glucose uptake regardless of insulin signaling. This pathway ensures a continuous supply of glucose to sustain basic cellular functions even in the absence of insulin stimulation.

Several studies have investigated the effect of PPs on the modulation of glucose uptake in mature adipocytes. In this review, eight studies reported a significant modulation of glucose consumed by cells after treatments with different extracts [10, 51, 53] and phenolic compounds [10, 11, 17, 19, 25, 27]. Two studies testing extracts reported an increase in insulin-independent glucose uptake [6, 49] while a single study showed a decrease in insulin-dependent glucose uptake [51]. Regarding single compounds, six studies evidenced an increase in adipocytes’ insulin-independent glucose uptake after treatments with flavanols [17], flavones [25], and flavan-3-ols [10], while an increase in the insulin-dependent glucose uptake was evidenced by flavonols [11]. Finally, two studies showed a decrease in insulin-dependent glucose uptake after treatments with flavones [27], and flavanones [19].

### Cellular Metabolic Markers Modulated by (poly)phenols

PPs have been widely investigated for their ability to modulate cellular markers associated with lipid and glucose metabolism, thermogenesis, and mitochondrial biogenesis. These bioactive compounds interact with multiple signaling pathways, leading to diverse metabolic outcomes.

Table 3 summarizes molecular findings and key cellular outcomes from studies on single compounds and (poly)phenol subclasses.

These effects are regulated by several regulatory proteins, organized in a hierarchy from general switches or more pathway-specific factors. In the next paragraphs, gene names are reported using standardized NCBI (Gene ID) nomenclature to ensure consistency and clarity, rather than relying on the original naming conventions used in the referenced studies. Due to reader-friendly communication, we have used mainly gene and protein abbreviations rather than full names.

Table 4 contains further information on the full names, commonly used aliases and NCBI Gene IDs.

**Table 3:** Summary of main (poly)phenolic compounds studied for their effects on adipocyte metabolism

Compound and (poly)phenol subclass	Molecular formula	Main adipocyte marker(s) modulated	Key cellular outcomes	Ref
Quercetin (Flavonol)	C15H10O7	↑ pAKT, ↓ Lpl, Dgat1/2, Cebpa, Slc2a4 (Glut4), Casp3	↑ lipolysis, ↓ lipogenesis	[11, 14, 15]
Chlorogenic Acid (Phenolic acid)	C16H18O9	↑ LPL activity	↑ lipid clearance	[46]
Resveratrol (Stilbene)	C14H12O3	↓ PPARγ, C/EBPα; ↑ SIRT1, pAMPKα	↑ lipolysis, ↑ mitochondrial function	[35, 38, 42, 43]
Naringenin (Flavanone)	C15H12O5	↑ Cpt1, Lipe, Pnpla2, pAMPK; Adipoq; Ucp2	↑ mitochondrial number / thermogenesis	[10, 18, 19]
Theaflavin-3,3′-digallate (Flavan-3-ol)	C43H32O20	↑ Ucp1	↑ mitochondrial number / thermogenesis	[29]
Acacetin (Flavone)	C16H12O5	↑ pPKA, pHSL; PRDM16-PGC1α-UCP1 signaling	↑ thermogenesis	[24]

**Table 4:** Full gene names, common aliases, and NCBI Gene IDs of the molecular markers discussed

Abbreviations (aliases)	Name (aliases)	GeneID
*Acsl1*	acyl-CoA synthetase long-chain family member 1	14,081
*Adipoq*	adiponectin	11,450
*Bcl2*	B cell leukemia/lymphoma 2	12,043
*Casp3*	caspase 3	12,367
*Cebpa*	CCAAT/enhancer binding protein alpha	12,606
*Cpt1a*	carnitine palmitoyltransferase 1a, liver	12,894
*Cpt1b*	carnitine palmitoyltransferase 1b, muscle	12,895
*Dgat1*	diacylglycerol O-acyltransferase 1	13,350
*Dgat2*	diacylglycerol O-acyltransferase 2	67,800
*Fasn*	fatty acid synthase	14,104
*Hsd11b1*	hydroxysteroid 11-beta dehydrogenase 1	15,483
*Lipe* (*Hsl*)	lipase, hormone sensitive	16,890
*Lep*	leptin	16,846
*Lpl*	lipoprotein lipase	16,956
*Nrip1* (Rip140)	nuclear receptor interacting protein 1	268,903
*Pgc1a*	peroxisome proliferator activated receptor, gamma, coactivator 1 alpha	19,017
*Plin1*	perilipin 1	103,968
*Pnpla2* (Atgl)	patatin-like phospholipase domain-containing protein 2 or adipose triglyceride lipase	66,853
*Ppara*	peroxisome proliferator activated receptor alpha	19,013
*Pparg*	peroxisome proliferator activated receptor gamma	19,016
*Rbp4*	retinol binding protein 4	19,662
*Sirt1*	sirtuin 1	93,759
*Slc2a4* (Glut4)	solute carrier family 2 member 4	20,528
*Tnfa*	tumor necrosis factor	21,926
*Tfap2a* (aP2)	transcription factor AP-2, alpha	21,418
*Trp53*	transformation related protein 53	22,059

#### Regulation of Lipid and Glucose Metabolism

**Fig. 2:**
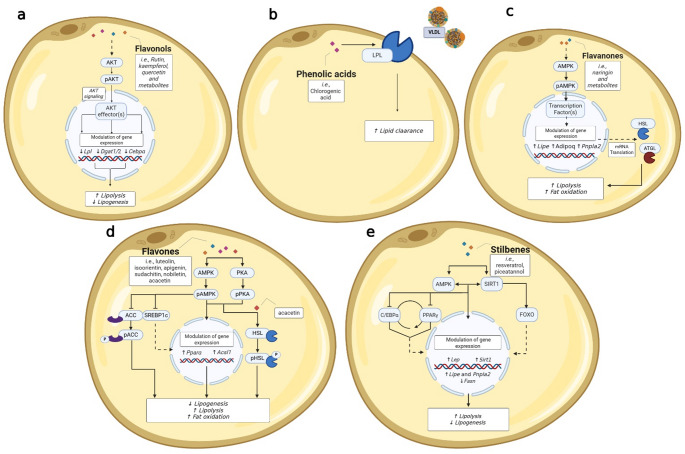
Overview of cellular metabolic markers involved in lipid and glucose metabolisms modulated by (poly)phenols, with a proposed reconstruction of the underlying molecular pathways. The figure summarizes the effects of different classes of (poly)phenols on key metabolic markers in 3T3-L1 adipocytes. The proposed pathways are reconstructed based on reported molecular modulations. (**a**) Flavonols modulate lipid metabolism by enhancing lipolysis and reducing lipogenesis; (**b**) Phenolic acids contribute to improved lipid clearance; (**c**) Flavanones are associated with enhanced lipolysis and increased fatty acid oxidation; (**d**) Flavones regulate lipid metabolism by decreasing lipogenesis while promoting lipolysis and fatty acid oxidation; (**e**) Stilbenes promote lipolysis and suppress lipogenesis

Several studies have investigated the mechanisms through which PPs influence adipogenesis, lipogenesis, and glucose homeostasis in mature 3T3L1 adipocytes (Table 4; Fig. 2). Flavonols have been reported to increase protein levels of pAKT [11] and mRNA levels of *Trp53*, *Bcl2*, and decrease mRNA levels of *Lpl*, *Dgat1*, *Dgat2*, *Cebpa* as well as *Slc2a4* (*Glut4*), *Casp3*, *Pnpla2* and *Lipe* [14, 15]. Flavones have been shown to increase protein levels of phosphorylated adenosine monophosphate-activated protein kinase (AMPK) in adipocytes [25, 27] and phosphorylated acetyl-CoA carboxylase (ACC) [27], while reducing sterol regulatory element-binding protein 1c (SREBP1c) and phosphorylated IRS [27]. The effects of flavones on phosphorylated protein-kinase A (PKA) and hormone-sensitive lipase (HSL) appear to vary depending on the specific compound. Kang et al. [27] reported a reduction in these markers following treatment with sinensetin. In contrast, pHSL levels were increased by acacetin [24], while sudachitin, nobiletin, and acacetin enhanced pPKA levels [23, 24]. Similarly, phosphorylated AKT was found to decrease after sinensetin treatment [27] but increased following exposure to isoorientin [25]. Finally, flavones have been reported to promote *Ppara* and *Acsl1* gene expression [26] which may contribute to increased lipid oxidation and reduced lipogenesis. Anthocyanins have been shown to increase mRNA levels of *Adipoq* and the adiponectin protein level [30]. Regarding flavanones, several studies have investigated the molecular mechanism exerted by naringenin and its derivatives. Overall, the experiments demonstrated that they can positively modulate *Lipe* and *Pnpla2* mRNA levels and their corresponding protein products [18], and pAMPK protein levels [10]. Additionally, another study reported the ability of naringenin to up-regulate *Adipoq* [19], supporting its modulation on lipolysis and fatty acids oxidation. Concerning mechanisms of action related to phenolic acids, Wang et al. [46] showed the ability of chlorogenic acid in increasing lipoprotein lipase (LPL) activity. In addition, another study investigated the effect of physiological doses of protocatechuic acid observing any effect on carnitine palmitoyltransferase 1 (CPT1) levels and activity [45]. Different stilbenes were investigated for their mechanisms: piceatannol positively modulates *Lep* at the transcriptional level, which encodes leptin which plays a major role in energy metabolism and satiety [36]. Resveratrol has been shown to increase SIRT1 and to reduce PPARγ and C/EBPα protein levels [38]. Moreover, resveratrol has been reported to be able to relieve lipolysis defects via reactivation of the SIRT1-dependent signaling pathway, restoring SIRT1, FOXO and ATGL protein levels [35]. Again, other studies showed the effectiveness of resveratrol and its metabolites to up-regulate pAMPKα and down-regulate pAKT protein levels [28], and to increase gene expression of *Pnpla2* [42, 43], *Cpt1b*, *Sirt1*, and *Lipe*, while decreasing *Fasn* [42]. Interestingly, different effects were evidenced by two resveratrol metabolites at the level of *Sirt1* expression depending on the position of the glucuronide group, with an increase induced by trans-resveratrol-4-O-glucuronide and a decrease prompted by trans-resveratrol-3-O-glucuronide [42].

#### Regulation of Thermogenesis and Mitochondrial Biogenesis

**Fig. 3:**
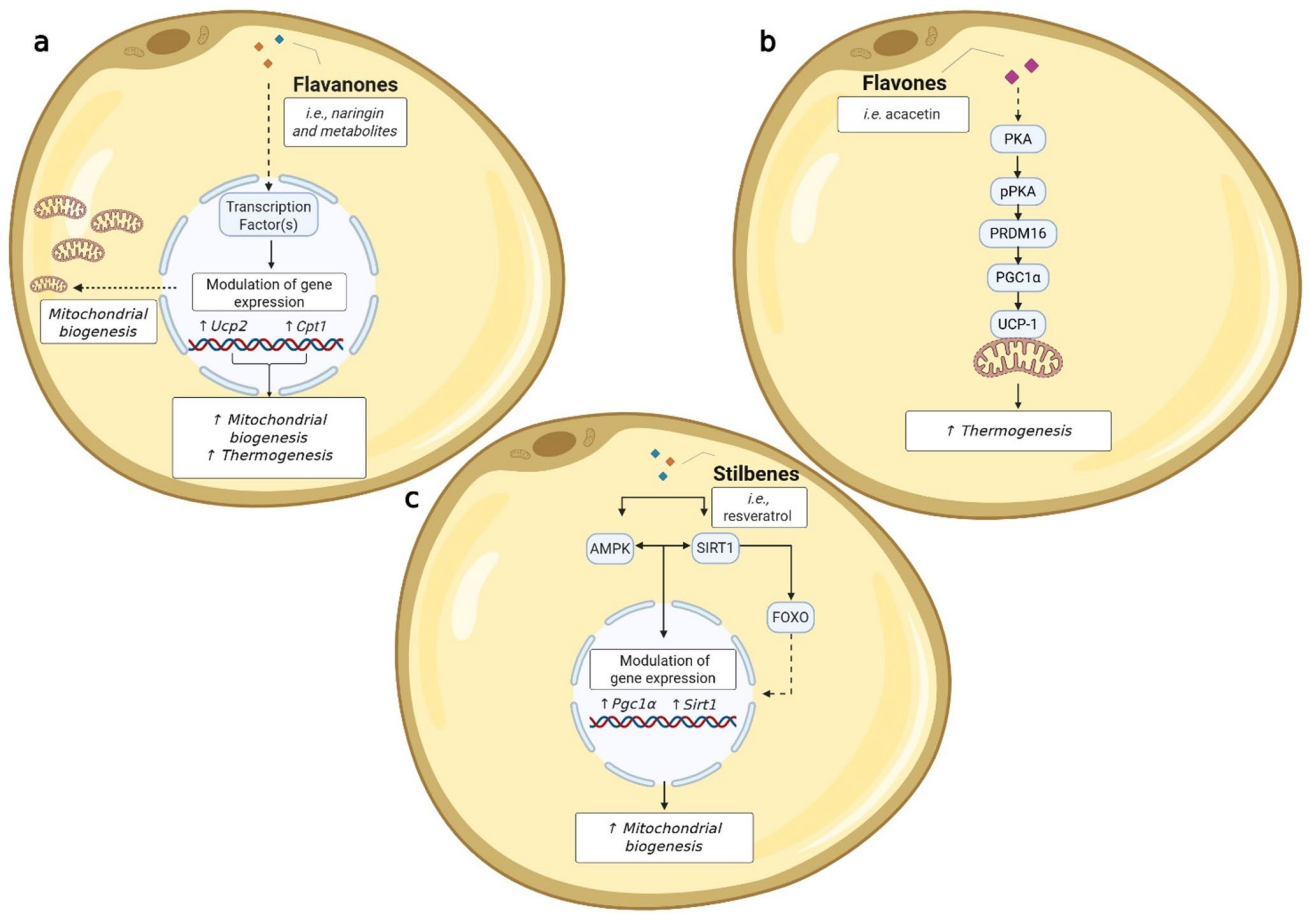
(**a-c**): Overview of cellular metabolic markers involved in thermogenesis and mitochondrial biogenesis modulated by (poly)phenols, with a proposed reconstruction of the underlying molecular pathways. The figure summarizes the effects of different classes of (poly)phenols on key metabolic markers in 3T3-L1 adipocytes. The proposed pathways are reconstructed based on reported molecular modulations. (**a**) Flavanones are associated with enhanced fatty acid oxidation, by stimulating mitochondrial biogenesis and thermogenesis: (**b**) Flavones regulate lipid metabolism by promoting the thermogenesis; (**c**) Stilbenes stimulate mitochondrial biogenesis and function, thereby supporting enhanced lipolysis

Among the various classes of PPs, flavanone, flavones, flavan-3-ols, and stilbenes have demonstrated the ability to enhance thermogenesis and/or mitochondrial biogenesis in mature 3T3L1 adipocytes (Table 4; Fig. 3). Naringin, belonging to the flavanone group, was proven to increase the mitochondria number in mature adipocytes [18]. In the same study, naringin was found to increase Cpt-1 and *Ucp-2* mRNA levels, highlighting its potential role in thermogenic pathways. Among flavones, acacetin has been shown to activate the PRDM16-PGC1α-UCP1 signaling pathway [24]. Within the stilbene subgroup, several studies have suggested that resveratrol acts as a modulator of mitochondrial biogenesis, likely through the activation of key regulators including SIRT1, pAMPKα, FOXO, and *Pgc1α*, whose expression or activity has been consistently upregulated in response to resveratrol treatment [28, 35, 42]. Finally, the flavan-3-ol theaflavin-3,3′-gallate was effective in upregulating *Ucp1* mRNA [29]. Moreover, in two studies, different PP-rich extracts have been demonstrated to increase *Ucp3* gene expression [55, 56].

#### Mechanistic Insights into (poly)phenol Actions on the Regulation of Metabolism

Based on the evidence summarized above, PPs exert their metabolic effects through distinct, yet interconnected mechanisms, primarily involving (1) the regulation of lipid and glucose metabolism, (2) the modulation of adipogenesis and lipolysis, and (3) the enhancement of thermogenesis and mitochondrial biogenesis. Modulation of the regulation of these pathways, as observed in mature 3T3-L1 adipocytes, can vary by phenolic compound class and contribute to metabolic homeostasis and may have implications for obesity and associated disorders (Figs. 2 and 3).

1. **Regulation of Lipid and Glucose Metabolism**: Flavonols modulate AKT signaling, promoting AKT phosphorylation and downregulating key lipogenic and adipogenic stimulating genes, such as *Lpl*, *Dgat1/2*, *Glut4*, *Casp3*, *Cebpa*, and *Pnpla2*. These molecular changes promote lipolysis while reducing lipid accumulation in adipocytes. Similarly, flavones activate AMPK and ACC phosphorylation while suppressing lipogenic regulators including SREBP1c and IRS, shifting cellular metabolism toward increased fatty acid oxidation and reduced lipid synthesis. Their effects on phosphorylated PKA and HSL appear to be compound-specific (e.g., acacetin, sudachitin, and nobiletin enhancing activation, whereas sinensetin reduces it). Additionally, flavones promote *Ppara* and *Acsl-1* gene expression, further supporting fatty acid oxidation. Among flavanones, naringenin and its derivatives enhance the expression of *Cpt1*, and protein levels of HSL, ATGL, and pAMPK, facilitating lipid mobilization and β-oxidation. Furthermore, flavanones increase the adiponectin-encoding gene (*Apipoq*), which may contribute to improved insulin sensitivity. Regarding phenolic acids, chlorogenic acid increases LPL activity, promoting lipid clearance and reducing circulating triglycerides. Although data on anthocyanins remain limited, they appear to enhance adiponectin expression, suggesting potential improvements in insulin sensitivity and glucose metabolism.
2. **Modulation of Adipogenesis and Lipolysis**: Several PPs influence adipogenesis and lipid mobilization through the regulation of SIRT1 and AMPK signaling pathways. Stilbenes, resveratrol and piceatannol suppress adipogenesis by downregulating PPARγ and C/EBPα, key transcription factors involved in adipocyte differentiation. Resveratrol restores SIRT1-FOXO-ATGL signaling, counteracting lipolysis defects, and enhances mitochondrial function by increasing pAMPKα while reducing pAKT levels. Additionally, resveratrol and its metabolites promote the gene expression of *Pnpla2*, *Cpt1b*, *Ppc1a*, *Sirt1*, and *Lipe*, while reducing *Fasn*, further favoring lipid mobilization and oxidation.
3. **Enhancement of Thermogenesis and Mitochondrial Biogenesis**: Several PP classes contribute to the stimulation of thermogenesis and mitochondrial biogenesis, suggesting potential benefits for energy expenditure and metabolic flexibility. Flavanones, particularly naringin, increase mitochondrial content in mature adipocytes and upregulate *Ucp2* expression, reinforcing thermogenic pathways. Similarly, flavones such as acacetin activate the PRDM16-PGC1α-UCP1 signaling cascade, a key pathway regulating brown adipocyte differentiation and thermogenesis. The stilbene resveratrol modulates the AMPK–SIRT1 signaling pathway, leading to the activation of *Pgc1α* and, consequently, an increase in mitochondrial biogenesis. Flavan-3-ols, including theaflavin-3,3′-digallate, promote *Ucp1* expression in adipose tissue.

Together, these findings highlight the complex and multifaceted roles of PPs in metabolic regulation, suggesting potential applications for obesity management and metabolic health improvement. However, further studies are required to elucidate the specific molecular targets and dose-dependent effects of these bioactive compounds.

## Conclusions

In conclusion, this systematic review highlights the significant anti-obesogenic potential of PPs on mature 3T3-L1 adipocytes, focusing on their effects on lipid and glucose metabolism, thermogenesis, and mitochondrial biogenesis. PPs, including single compounds and PP-rich extracts, were shown to reduce intracellular lipid content and enhance lipolytic activity, particularly among flavonols, flavones, flavanones, flavanonols, phenolic acids, and stilbenes. PPs have also shown to be able to modulate glucose uptake, with some increasing insulin-dependent and insulin-independent glucose uptake and others inhibiting it. Key mechanisms involved included activation of AMPK, increased adiponectin levels, and modulation of genes such as *Ppara*, *Cpt1*, and *Sirt1*. Additionally, PPs promoted thermogenesis and mitochondrial biogenesis, evidenced by increased expression of UCPs and a higher number of mitochondria in adipocytes.

These findings suggest that PPs hold promise as anti-obesity agents. However, animal studies and clinical trials are needed to confirm their effects observed *in vitro*. Additionally, further research is needed to fully understand their mechanisms and identify the most effective types and concentrations to use. The demonstration of such effects could bring in the future the development of dietary recommendations aimed at promoting the consumption of PP-rich foods with lipid-lowering properties.

## Supplementary Information

Below is the link to the electronic supplementary material.

Supplementary Material 1

Supplementary Material 2

## Data Availability

No datasets were generated or analysed during the current study.

## Key References

- Green H, Meuth M. An established pre-adipose cell line and its differentiation in culture. Cell. 1974; 3:127–33. 10.1016/0092-8674(74)90116-0.

The differentiation of 3T3-L1 preadipocytes into adipocytes is a regulated process.

- Rendine M, Venturi S, Marino M, Gardana C, Møller P, Martini D, et al. Effects of Quercetin Metabolites on Glucose-Dependent Lipid Accumulation in 3T3-L1 Adipocytes. Molecular Nutrition & Food Research 2025; 0:e70070. 10.1002/mnfr.70070.

Relevance of studying bioactive compounds in fully differentiated adipocytes.

- Collins JM, Neville MJ, Pinnick KE, Hodson L, Ruyter B, van Dijk TH, et al. De novo lipogenesis in the differentiating human adipocyte can provide all fatty acids necessary for maturation. J Lipid Res. 2011; 52:1683–92. 10.1194/jlr.M012195.

Adipocytes exhibit intrinsic metabolic plasticity during differentiation.
