# Supplementary material for: Effect of (Poly)phenols on Lipid and Glucose Metabolisms in 3T3-L1 Adipocytes: an Integrated Analysis of Mechanistic Approaches
Source: Curr Obes Rep. 2025 Aug 6;14(1):64. doi: 10.1007/s13679-025-00656-6 (PMC12328513; doi:10.1007/s13679-025-00656-6)
Supplement: Supplementary file 1 — Supplementary Material 1 [file 13679_2025_656_MOESM1_ESM.docx]

**Supplementary file 1**: Search strategy following PRISMA guidelines^1^.

PubMed

1. 3T3-L1 [AllFields]
2. 3T3-L1 adipocytes [AllFields]
3. 1 OR 2
4. polyphenols [All Fields]
5. phenolic compounds [All Fields]
6. flavonoids [All Fields]
7. 4 OR 5 OR
8. lipogenesis [All Fields]
9. adipogenesis [All Fields]
10. anti-obesity [All Fields]
11. anti-adipogenic [All Fields]
12. lipid accumulation [All Fields]
13. obesity [All Fields]
14. 8 OR 9 OR 10 OR 11 OR 12 OR 13
15. 2 AND 6 AND 14

Scopus

1. TITLE-ABS-KEY (3T3-L1)
2. TITLE-ABS-KEY (3T3-L1 AND adipocytes)
3. 1 OR 2
4. TITLE-ABS-KEY (polyphenols)
5. TITLE-ABS-KEY (phenolic AND compounds)
6. TITLE-ABS-KEY (flavonoids)
7. 4 OR 5 OR 6
8. TITLE-ABS-KEY (lipogenesis)
9. TITLE-ABS-KEY (adipogenesis)
10. TITLE-ABS-KEY (anti-obesity)
11. TITLE-ABS-KEY (anti-adipogenic)
12. TITLE-ABS-KEY (lipid AND accumulation)
13. TITLE-ABS-KEY (obesity)
14. 33 OR 34 OR 35 OR 36 OR 37 OR 38 OR 39 OR 40 OR 41 OR 42 OR 43
15. LIMIT-TO LANGUAGE (English)
16. 2 AND 6 AND 14 AND 15

**Web of Science (**https://www.webofscience.com/wos/woscc/summary/c4a3a0c5-fad2-407b-a740-b5e5ced605d4-8938a596/relevance/1)

1. 3T3-L1 [AllFields]
2. 3T3-L1 adipocytes [AllFields]
3. 1 OR 2
4. polyphenols [All Fields]
5. phenolic compounds [All Fields]
6. flavonoids [All Fields]
7. 4 OR 5 OR
8. lipogenesis [All Fields]
9. adipogenesis [All Fields]
10. anti-obesity [All Fields]
11. anti-adipogenic [All Fields]
12. lipid accumulation [All Fields]
13. obesity [All Fields]
14. 8 OR 9 OR 10 OR 11 OR 12 OR 13
15. 2 AND 6 AND 14

1. Liberati A, Altman DG, Tetzlaff J, et al. The PRISMA statement for reporting systematic reviews and meta-analyses of studies that evaluate health care interventions: explanation and elaboration. J Clin Epidemiol. 2009;62:e1000100.
