# Supplementary material for: Effect of (Poly)phenols on Lipid and Glucose Metabolisms in 3T3-L1 Adipocytes: an Integrated Analysis of Mechanistic Approaches
Source: Curr Obes Rep. 2025 Aug 6;14(1):64. doi: 10.1007/s13679-025-00656-6 (PMC12328513; doi:10.1007/s13679-025-00656-6)
Supplement: Supplementary file 2 — Supplementary Material 2 [file 13679_2025_656_MOESM2_ESM.docx]

| **First author**  **and year of**  **publication**  **(ref)** | **Seeding conditions** | **Cell culture conditions** | **Cytotoxicity assay** | **Treatment** | **Control used** | **Duration of treatment** | **Assays** | **Main findings** | **Class** |
| --- | --- | --- | --- | --- | --- | --- | --- | --- | --- |
| Gómez-López – 2024 [47] | na | DMEM (glucose na) supplemented with 10% FBS and 1% penicillin/streptomycin (10.000 U/mL) | Crystal violet staining | Whole fruit, peel, pulp, or bagasse (BA) extracts from *O. stricta var. dillenii* (10, 25, 50 and  100μg/mL) on day 8 after differentiation | Untreated cells | 24 h | TG and protein content  qPCR  Western blot | Various extracts of *O. stricta var. dillenii* (50-100μg/mL):  ↓ TG content  ↑ pACC and pHSL protein levels | Extract |
| Kobayashi – 2024 [28] | Cells seeded at density of 3x10^4^ cells/well in 24‑well plate | DMEM (glucose na) supplemented with 10% FBS and antibiotic/antimycotic | na | Prenylflavonoids-rich extracts of *Lespedeza* spp (0.00001, 0.0001, 0.001, 0.01mg/ml) after differentiation | Untreated cells and  pioglitazone (0.28, 2.8,  28, 280μM) as positive control | 96 h | Oil red O staining  ELISA | *Lespedeza* extracts (0.00001‑0.001mg/ml):  ↑ lipid accumulation  ↑ adiponectin protein levels | Extract |
| Wang – 2024 [35] | Cells seeded at density of 5 × 10^4^ cells/mL in 6-well plates | DMEM (glucose na) supplemented with 10% BCS and 1% antibiotic–  antimycotic | na | Resveratrol (50μM) with SIRT1 inhibitor (10µM)  Or FOXO1 inhibitor (1µM) | Untreated cells | 2 h pre-treatment with Resveratrol  24 h treatment with inhibitors | NAD+/  NADH assay  qPCR  Western blot  ChIP assay | Resveratrol pre-treatment (50μM):  Restore NAD+/  NADH ratio  Restore FOXO1 and *Atgl* gene expression | Stilbene |
| Xiang – 2024 [45] | na | DMEM supplemented with 10% FBS, 1% penicillin/streptomycin | na | Protocatechuic acid (PCA; 0.5 and 5μM) after differentiation in both white- like adipocytes and brown-like adipocytes | Untreated cells | 24 h | Western blot  qPCR  CPT1enzymatic Activity  Fat acids oxidation | PCA (0.5 and 5μM):  = *Cpt1* gene expression, protein levels, and enzymatic activity  = fat acids oxidation | Phenolic acids |
| Abe – 2023 [23] | na | DMEM (glucose na) supplemented with 10% FBS and 1% penicillin/streptomycin  mixed solution | LDH Activity Assay | Sudachitin and nobiletin (30 and 50µM) on day 8 after differentiation | DMSO 0.1%  Isoproterenol (10µM) as positive control | 0-120 m (for Western blot)  24 and 48 h | Oil red O staining  Glycerol release  cAMP assay  Western blot | Sudachitin (50 µM):  ↓ Lipid accumulation  Sudachitin and Nobiletin 50µM:  ↑ Glycerol release and cAMP levels  Sudachitin and Nobiletin 30µM:  ↑ pPKA substrates and pHSL protein levels | Flavone |
| Asmara – 2023 [10] | Cells seeded at density of 3 x 10^3^ cells/mL in 96-well plate | DMEM (high glucose) supplemented  With 9% BCS and 1% penicillin, streptomycin glutamine | MTT | Flower, leaf and bark extracts of *Acacia saligna* (12.5 and 50µg/mL); Naringenin, naringenin-7-O-L-arabinopyranoside,  isosalipurposide, quercitrin, epicatechin, and myricitrin (0.5 and 10µM) | Vehicle  Insulin (100µM) and  metformin (10µM) as positive controls | 30 m (glucose uptake)  24 h (other assays) | Glucose uptake  Western blot  Mitochondrial Membrane Potential (MMP) Measurement | Flower and Leaf extracts (50µg/mL) and (-)-epicatechin (10µM):  ↑ Glucose uptake  Naringenin, naringenin-7-O-L-arabinopyranoside,  epicatechin (10µM):  ↑ MMP  Flower extract (50µg/mL) and naringenin-7-O-L-arabinopyranoside,  isosalipurposid (10µM):  ↑ pAMPK protein levels | Extract  Flavanone  (Naringenin)  Flavonol  (Isosalipurposide, Quercitrin, Myricitrin)  Flavan-3-ol  (Epicatechin) |
| Cheng – 2023 [49] | na | DMEM (high glucose) supplemented with 9% NBCS, NaHCO3 (1.5 g/L) and 1% penicillin-streptomycin | na | MGF-1-7 solutions containing mixed extract of bainikuekisu, Prunus mume, black garlic, and Hsian-tsao (10, 25, 50, 100, and 250µg/mL) on day 7 after differentiation | na | 48 h | Oil red O staining  Intracellular TGs  GPDH Activity | Various MGF solutions (10-250 μg/mL):  ↓ lipid accumulation  ↓ TGs content  ↓ GPDH activity  MGF 3-7 at 10-250 μg/mL:  ↓↓ lipid accumulation  ↓↓ TGs content  ↓↓ GPDH activity | Extract |
| Pérez-Ramírez – 2023 [50] | Cells seeded at density of 10⁴ cells/well | DMEM (glucose na) | na | Polyphenol-rich extract of Azufrasin variety (PP-AZ) and polyphenol-rich extract of FJ Dalia variety (PP-FJD) (150μg/mL) after differentiation | DMSO | 30 m | qPCR | PP-AZ and  PP-FJD (150μg/mL):  ↓ PPARγ, CEBPα, FASN and ACCα and ↑ *Cpt1a, Acadm*  *Glut4, Irs1* and *Pi3k* mRNA levels | Extract |
| Rakib – 2023 [36] | na | DMEM (glucose na)  supplemented with,  L-glutamine (2 mM), sodium pyruvate (1 mM), penicillin/streptomycin (100 U/mL/100 μg/mL) and 10 % FCS | na | Piceatannol (10 μM) on day 7 after differentiation | Untreated cells | 24 h | Cell morphology (cell area, perimeter, and circularity)  Oil Red O staining  qPCR | Piceatannol (10 μM):  ↓ Cell size  ↓ Lipid accumulation  ↑ leptin mRNA levels | Stilbene |
| Wang – 2023 [18] | Cells were seeded in 96 well plate | DMEM (glucose na) supplemented with 10% FBS | LDH assay | Naringin (100µg/mL) on day 7 after differentiation | na | 4 d | Oil red O staining  Mito-tracker green staining  qPCR  Western blot | Naringin (100µg/mL):  ↓ Lipid accumulation  ↑ Mitochondria number  ↑ *Hsl, Atgl, Cpt1* and *Ucp2* mRNA levels  ↑ pHSL and ATGL protein levels | Flavanone |
| Zhang – 2023 [24] | na | H-DMEM supplemented with 10% (w/v) FBS and 1% (w/v) penicillin–streptomycin | ns | Acacetin (ACA; 20 and 40μM) after differentiation | Untreated cells and cells treated with Adenyl cyclase inhibitor (13μM) or beta-adrenergic receptor blocker (5μM) | 24 h | Western blot  ELISA | ACA (20 and 40μM):  ↑ cAMP levels  ↑ PRDM16, PGC1α, UCP1, and  PKA protein levels | Flavone |
| Lee – 2022 [51] | na | DMEM (glucose na) containing 10% FBS | MTT assay | Sorghum extracts (different genotypes PI570481, SC84, Sumac and white) (1.25, and 2.5mg/mL) after differentiation | White sorghum extract | 24 and 48 h | Glucose uptake  Glycerol release  Western blot | Sorghum extracts (2.5mg/mL):  ↓ insulin-dependent glucose uptake  Sorghum extracts (1.25 and 2.5mg/mL):  = glycerol release  ↓ insulin-independent glucose uptake  ↓ IRS-1 and pAKt and  = HSL protein levels | Extract |
| Wu – 2022 [22] | Cells seeded in 96-well plates (7 × 10³ cells/well) and 24-well plates (3.5 × 10⁴ cells/well) | DMEM (low glucose) containing 10% FBS and 1% penicillin-streptomycin solution | MTT assay | Ampelopsis grossedentata (AG) and ampelopsin (10 and 30µg/mL) on day 10 after differentiation | DMSO | 48 h | Oil red O staining | AG (10 and 30µg/mL):  ↓ lipid accumulation | Extract  +  Flavanonol |
| Kowalska – 2021 [34] | Cells seeded in 12-well plates at density of 2.5x10⁴ cells/well | DMEM (glucose na) supplemented with 10% FBS | MTT assay | Lingonberry fruit ACN and PP fractions (5, 10, and 20µg/mL) after differentiation | Untreated cells | 24 h | Oil red O staining  Intracellular TG  qPCR  ELISA | PP (5-20µg/mL):  ↓ lipid accumulation and TG content  ↓ *Lep* mRNA and protein levels  PP (10-20µg/mL):  ↓ *Tfap2a* and *Dagt1* gene expression  ↑ adiponectin mRNA and protein levels  PP (20µg/mL):  ↓ *Fas* mRNA levels  ACN (10-20µg/mL):  ↓ lipid accumulation  ↓ TG content  ↑ adiponectin mRNA and protein levels  ACN (20µg/mL):  ↓ *Fas*, *Tfap2a* and *Dagt1* gene expression  ↓ leptin mRNA and protein levels | Extract  Anthocyanins |
| Lim – 2021 [11] | Cells seeded in 96-well plates at density of 10⁴ cells/well | DMEM (high glucose) supplemented with 10% FBS | MTT assay | Rutin and quercetin-3-O--D-glucoside (Q3G) (5 and 10μM) on day 8 after differentiation | Untreated cells | 24 h | Oil red O staining  Glucose uptake  Western blot | Rutin and Q3G (5 and 10μM):  = lipid accumulation  ↑ insulin-dependent glucose uptake  Rutin (10 μM) and Q3G (5, 10 μM):  ↑ pAkt protein levels | Flavonol glycoside |
| Muramatsu – 2020 [12] | na | DMEM (glucose na) supplemented with 10% FBS | Cell Counting Kit-8 assay | Larix kaempferi extract (LK-ME) diluitions (100, 200, 400 and 800 containing taxifolin at 102,5, 51.3, 25.6 and 12.8μM, respectively) and taxifolin, quercetin, and luteolin (75, 150, 300 and 600μM) after differentiation | Untreated cells as negative control and berberine chloride (0.1-2 μg/mL) as positive control | 3 d | Oil red O staining | LK-ME diluitions (100 and 200):  ↓ lipid accumulation  Taxifolin (150-600μM) and Quercetin (600μM):  ↓ lipid accumulation | Extract  Flavonol (quercetin)  Flavanonol (taxifolin)  Flavone  (luteolin) |
| Peng – 2020 [52] | Cells seeded in 6 well-plates at density of 1 × 10⁶ cells/well | DMEM (high glucose) supplemented with 10% BCS, 1% penicillin/streptomycin, 1.5 g L−1 sodium bicarbonate, 2 mM l-glutamine and 1 mM sodium pyruvate | MTT assay | Solanum nigrum L. extract (SWE) and its derived polyphenols (SNPE) (0.3, 0.4 and 0.5mg/ml) on day 12 after differentiation | na | 24 h | Oil red O staining  Western blot | SNPE (0.3-0.5mg/ml):  ↓ lipid accumulation  ↓ FaS, SREBP-1, HMG-CoR, SREBP-2 and  ↑ pAMPK, CPT-1 and PPARα protein levels | Extract  PP fraction |
| Ziqubu -2020 [25] | Cells seeded in 96-well plates at a density of 10,000 cells/well | DMEM (glucose na) supplemented with 10% FBS | MTT assay | Isoorientin (0.001, 0.01, 0.1, 1, 10, and 100µM) on day 8 after differentiation | Untreated cells and positive controls (isoproterenolCL-316,2431, insulin, metformin) | 4 h | Oil red O  Staining  Intracellular ATP  Glucose uptake  Glycerol release  Seahorse test  Western blot | Isoorientin (0.1 and 1µM):  ↓ lipid accumulation  ↑ OCR%  Isoorientin (0.1-100µM):  ↑ glucose uptake  ↑ glycerol release  ↑ pATK and pAMPK protein levels  Isoorientin (0.001, 0.1 and 10µM):  ↑ ATP production | Flavone glycoside |
| Aranaz – 2019 [13] | 96 (5 × 103 cells/well) or in 12 well (7 × 104 cells/well) plates | DMEM (glucose na)  supplemented with 10% CBS and 1% penicillin/streptomycin | MTS assay | Apigenin, luteolin, hesperidin, naringin, resveratrol, curcumin, myricetin, kaempferol, quercetin, p-coumaric acid,  ellagic acid, ferulic acid, gallic acid and vanillic acid (10, 50 and 100µM) on day 8 after differentiation | DMSO (0.1%) | 8 d | Nile red staining | Quercetin and naringin (100µM) and  apigenin, luteolin, resveratrol, curcumin, myricetin, kaempferol (50-100µM) and  p-coumaric acid (50µM):  ↓ lipid accumulation | Flavone  (apigenin, luteolin)  Flavanone glycoside (hesperidin)  Flavanone (naringin)  Stilbene  (resveratrol)  Curcuminoids (curcumin)  Flavonol (quercetin, kampferol, myricetin)  Phenolic acids |
| Eseberri – 2019 [14] | 6-well plates | DMEM (glucose ns) with 10% FCS, 1%  Penicillin/Streptomycin (10,000 U/mL) | na | Quercetin (Q), isorhamnetin (ISO), tamarixetin (TAM),  quercetin-3-O-glucuronide (3G), quercetin-3-O-sulfate (3S), and quercetin-4-O-sulfate (4S). Mixture (3S+4S) (0.1, 1 and 10µM) on day 12 after differentiation | Ethanol (0.095%) | 24 h | Intracellular TGs  Lipolysis assay  qPCR | Q (10 µM), 3S (1-10µM) and 3S+4S (10µM):  ↓ TGs content  3S (10µM):  ↓ *Lpl*, *Dgat1*, *Dgat2*, *Glut4*, *Cas3* and  ↑ *Trp53*, *Bcl2* mRNA levels  3S+4S (10µM):  ↑ *Trp53* mRNA levels | Flavonol |
| Zielinska-Wasielica – 2019 [53] | Cells seeded in 24-well plates | DMEM (glucose na) supplemented with 10% calf serum | MTT assay | Elderberry fruit extract (EDB) (5, 10, and 20mg/mL) after differentiation | Untreated cells | 24 h | Intracellular TG  Glucose uptake  qPCR  ELISA | EDB (dose dependently):  ↓ leptin mRNA and protein levels  EDB (5, 10, and 20mg/mL):  = TG content  ↑ glucose uptake  EDB (20mg/mL):  ↑ adiponectin mRNA and protein levels | Extract |
| Choe – 2018 [54] | na | DMEM (high glucose) supplemented with 10% BCS | MTT assay | Water‑extracted plum (WAP) (Prunus salicina L. cv. Soldam) (500 and 1000μg/ml) on day 10 after differentiation | Sulforaphane as a positive control | 24 h | Oil Red O staining  qPCR  Western blot analysis | WAP (500 and 1000μg/ml):  ↓ Lipid accumulation  ↑ AMPK, pAMPK and  ↓ pPI3K, pAkt SREBP1c, C/EBPα, PPARγ mRNA and protein levels. | Extract |
| Gartziandia – 2018 [37] | 6-well plates | DMEM supplemented with 10% FCS and 1% Penicillin/Streptomycin | na | Resveratrol (1 and 10µM) on day 12 after differentiation | na | 24 h | Intracellular TG | Resveratrol (1 and 10µM):  ↓ TG content | Stilbene |
| Modi – 2018 [38] | Cells seeded in 48-well plates at density of 2×10⁵ | DMEM (glucose na) supplemented with 10% FBS, 2 mM l-glutamine and 1% penicillin/streptomycin | Sulforhodamine B | Strigolactone analog (GR24) and pinosylvin (20 and 60μM) and resveratrol (60μM) on day 10 after differentiation | DMSO | 24 h | NAD+/NADH assay  Western blot | GR24 (60μM):  ↑ NAD+/NADH  ↑ SIRT1 protein levels  GR24, pinosylvin and resveratrol (60μM):  ↓ PPARγ and C/EBPα protein levels | Stilbene |
| Jack – 2018 [55] | Cells were seeded in 96- (4 × 103 cells/well), 24-  (2.0 × 104 cells/well), or 6- (6.0 × 104 cells/well) well plates | DMEM (high glucose) supplemented with 10% CS | ATP assay | Four fractions from a crude polyphenol-enriched  organic fraction of Cyclopia intermedia (1, 10, 50, 100μg/mL) on day 7 after differentiation. | Mangiferin 0.01 μM and isoproterenol 10 μM as positive controls | 24 h | Oil red O staining  Glycerol release  qPCR | F1 and F4 (1 and 10μg/mL),  F2 (100μg/mL), and  F3 (1-100 μg/mL):  ↓ lipid accumulation  F1-F3 (various concentrations):  ↑ glycerol release  F1 (10μg/mL) and F2 (100 μg/mL):  ↑ *Hsl* mRNA levels  F1 and F4 (10 μg/mL):  ↑ *Ucp3* mRNA levels | Extract |
| Torres Villareal – 2018 [15] | Cells seeded in 96-well plates at a density of 10,000 cells/well | DMEM (high glucose) supplemented with 10% (vol/vol) newborn calf serum, 100 units/ml penicillin, and 100 μg/ml streptomycin | CellTiter-Glo Luminescent | Kaempferol (60μM) on day 12 after differentiation | DMSO | 24 h and 9 d | q-PCR | Kaempferol (60μM):  ↓ *Cebpa*, *Pnpla2*, *Lipe* mRNA levels | Flavonol |
| Wang – 2018 [46] | na | DMEM (glucose na) supplemented with 10% fetal bovine serum | na | NTU 101-fermented tea (1, 25, 50, 75 and 100 ppm). Epigallocatechin gallate (EGCG) and chlorogenic acid (1, 25, 50, 75, 100, 200 and 250ppb) on day 8 after differentiation | Unfermented leaf extract | 4 d | Glycerol release  Heparin-releasable lipoprotein lipase (HR-LPL) activity assay | NTU 101-fermented tea, EGCG, and chlorogenic acid (various concentrations):  ↑ Glycerol release  EGCG and chlorogenic acid (50ppb):  ↑ HR-LPL activity | Extract  Flavan-3-ol  Phenolic acids |
| Zhu – 2018 [39] | Cells seeded at density of 3×10⁴ cells/well | DMEM (glucose na) supplemented with 15% FBS, 100 IU/mL penicillin, and 100 µg/mL streptomycin | na | Resveratrol (Res) (10, 25µM) on day 10 after differentiation | DMSO | 3 d | Oil red O staining  Western blot | RES (dose dependently):  ↓ lipid accumulation | Stilbene |
| Jack – 2017 [56] | Cells seeded in 96, 24 or 6 multi-well plates at 4 × 103,  2.0 × 104 or 6.0 × 104 cells/well, respectively | DMEM  (high glucose) supplemented with 10% NBCS, 100 U/mL  penicillin and 100 μg/mL streptomycin | MTT and ATP assay | Aqueous and organic fractions of Cyclopia spp (*C. maculata*, *C. intermedia* and *C. subternata*) (1, 10, 50, 100μg/mL) on day 7 after differentiation | Isoproterenol (10 μM)  as positive control | 24 h | Oil red O staining  qPCR | Aqueous fractions of *C. maculata* and C. *subternata*, and the organic fraction of C. intermedia (various concentrations):  ↓ lipid accumulation  The organic fraction of *C. intermedia* (various concentrations):  ↑ *Hsl*, *Ucp3* and *Pparg* mRNA levels | Extract |
| Kim – 2017 [57] | Cells seeded in 6 well plates | DMEM (glucose na) supplemented with 10% FBS | MTT or CCK-8 assay | Gelidium amansii -3-fraction (GAE) (10, 25, 50, 100 and 200µg/mL) on day 8 after differentiation | DMSO | 8 d | Oil Red O staining  Intracellular TG  Western blot | GAE (10-200µg/mL):  ↓ lipid accumulation  GAE (25- 200µg/mL):  ↓ TG content  GAE (50-200µg/mL):  ↑ p-ATGL protein levels  GAE (50–200µg/mL):  ↑ p-HSL protein levels  GAE (dose-dependently):  ↑ AMPK protein levels | Extract |
| Garcia-Diaz – 2016 [26] | Cells seeded in Petri dishes at density of 5x106/dish | DMEM (very high glucose) supplemented with 1 mM sodium pyruvate, 2 mM glutamine, 0.1 mM non-essential amino acids,  gentamicin and 10% FBS. | na | Tilianin (10µM) on day 8 after differentiation | Pioglitazone and fenofibrate as positive controls | 24 h | qPCR | Tilianin (10µM):  ↑ PPARα and *Acsl1* mRNA levels | Flavone |
| Chen - 2015a [28] | na | DMEM (glucose ns) with 10% FBS and supplemented with  100 U/mL of penicillin and 100 mg/mL of streptomycin. | na | Resveratrol (25, 50 and  100µM) when >90% cells  attained adipocyte morphology | na | 12 and 24 h | LDH release assay  Apoptosis assay  Mitochondrial membrane potential (MMP)  Western blot | Resveratrol (dose dependently):  ↑ LDH leaking ratio  ↓ MMP  ↑ p-AMPKα and  ↓ p-AKT protein levels | Stilbene |
| Chen - 2015b [40] | na | DMEM with 10%  FBS and penicillin-streptomycin. | na | Green tea catechins (GTCs) (2.3, 11.5 and 23µM) in the presence or absence of NE (0.1 or 1 mM) on day 10-12 after differentiation | Isoproterenol  10mM (positive control) | 6 and 24h | Glycerol and FFAs release  qPCR  Western blot | GTCs (dose dependently):  ↑ NE-induced glycerol and FFAs release  ↑ *Hsl*, *Atgl*, and Plin1 mRNA levels  ↑ NE-induced increase p-HSL protein levels | Favan-3-ol |
| García-Carrasco – 2015 [58] | 350 cell/well | DMEM with 10% FBS, 4-(2-hydroxyethyl)-1-piperazineethanesulfonic acid  buffer solution 25 mM, 1% glutamine, and 1% antibiotic-antimycotic mixture | MTT assay | Taraxacum officinale (dandelion) extracts (400 and 600μg/mL) on day 10 after differentiation | 0.2% DMSO | 48 h | Oil-Red-O Staining  Intracellular cholesterol | Dandelion extracts (400 and 600μg/mL):  ↓ cell viability  ↓ lipid accumulation  ↓ cholesterol content | Extract |
| Kang – 2015 [59] | Cells seeded in 96-well plates at density of 10⁴ cells/well | DMEM (glucose na) supplemented with 10% BCS and 1% penicillin/streptomycin. | MTT assay | Smilax china L. leaf ethanol extract (wsSCLE) (0,1 and 0,25mg/ml) on day 11 after differentiation | Untreated cells | 24 h | Glycerol release  cAMP assay  ELISA  Western blot | wsSCLE (dose dependently):  ↑ cAMP synthesis  wsSCLE (0,25mg/ml):  ↑ glycerol release  ↑ pPKA and p-HSL protein levels | Extract |
| Ko – 2015 [29] | na | DMEM supplemented with 10% FBS, 100 U/ml penicillin, and 100 µg/ml streptomycin | MTT assay | Theaflavin-3,3′-digallate (TF3) (25 and 50µM) on day 9 after differentiation | na | 48 h | Oil Red O staining  Intracellular TG  Glycerol and NEFA release  GPDH activity  Western blot  qPCR | TF3 (25-50µM):  ↓ GPDH activity  ↑ glycerol release  = NEFA release  ↑ *Ucp1* and *Hsl* mRNA levels  ↑ p-Akt and pACC protein levels  TF3 (25µM):  ↑ p-AMPK protein levels  TF3 (50µM):  ↓ lipid accumulation and TG content  ↑ *Lcad*, *Cpt-1*, *Ucp-2*, *Glut4*, mRNA levels | flavan-3-ol |
| Noriega-Gonzalez 2015 [41] | na | DMEM (glucose na) supplemented with15% NCS, 100 IU/mL penicillin, and 100 µg/mL streptomycin | na | Resveratrol (RSV) (25 and 50µM) on day 8 after differentiation | na | 48 h | Oil red O staining  Cell morphology    Mitotracker Green FM | Resveratrol RSV (25 and 50µM):  ↓ lipid accumulation and cell size  = mitochondrial number | Stilbene |
| Subramaniam – 2015 [60] | Cells seeded in 24-well plates | DMEM (glucose na) supplemented with 10% FBS | MTT assay | Fermented and unfermented ethanol extracts  of *Ganoderma* spp. mycelia (20 and 100µg/mL) after differentiation | 1% DMSO and epinephrine (1 µM) as positive control | 48 h | Glycerol release | Ethanol extract (100µg/mL):  ↓ glycerol release and epinephrine-induced glycerol release | Extract |
| Jeon – 2014 [16] | Cells seeded at density of 8×10⁴  cells/well | DMEM (glucose na) supplemented with 10 % BCS and penicillin–streptomycin (100 units/mL) | MTT assay | (-) Rutin and Rutin polymer fraction (RPF) (125, 250 and 500μM) on day 8 after differentiation | DMSO | 24 h | Intracellular TG  Glycerol release  GPDH activity    qPCR | (-) Rutin and RPF (dose dependently):  ↓ TG content  ↓ GPDH activity  (-) Rutin (250-500µM):  ↓ *Srebo1c*, *Cebpa*, *Fas* and *Adipoq* mRNA levels  RPF (125-500μM):  ↑ glycerol release  ↓ *Pparγ*, *Cebpa*, *Srebp1c*, *Fas* and  ↑ *Adipoq* mRNA levels | Flavonol glycoside |
| Okabe – 2014 [61] | na | DMEM (glucose na) supplemented with 10% FBS and 1% penicillin/streptomycin | MTT assay | Kaempferia parviflora extracts (KPE) (3, 10, 30mg/mL) and KPE  polymethoxyflavonoids (PMF) (3, 5, 10, 15, 30μM) on day 8 after differentiation | DMSO | 4 d | Oil red O staining  Intracellular TG  Glycerol release  qPCR  Western blot | KPE and PMF (various concentrations):  ↓ lipid accumulation  KPE (3 and 10mg/mL):  ↑ *Adipoq*, *Atgl* and *Hsl* mRNA levels  PMF (15 and 30μM):  ↑ glycerol release  ↑ Adiponectin, ATGL and HSL mRNA levels  PMF (15 and 30μM):  ↑ ATGL protein levels | Extract |
| Kang – 2013 [27] | Cells seeded in 12-well plates | DMEM (glucose na) supplemented with 10% BCS and 1% penicillin/streptomycin | LDH assay | Sinensetin (2, 10, 40µM) after differentiation | DMSO | 24 h | Glucose uptake  qPCR  Western blot | Sinensetin (40 µM):  ↓ insulin-dependent glucose uptake  ↑ *Cpt1a* mRNA levels  ↓ SREBP1c, pPKA, pHSL, pAkt, pIRS, and  ↑ pAMPK, pACC and protein levels | Flavone |
| Richard – 2013 [19] | Cells seeded in 6 well plates | DMEM (glucose na)  supplemented with  10% FBS | na | Naringenin (12, 25 and 50μg/mL) | DMSO | 24 and 48 h | Oil Red O  staining  Glucose uptake  Western blot | Naringenin (dose dependently):  ↓ insulin-dependent glucose uptake  Naringenin (50 μg/mL):  ↓ adiponectin mRNA levels | Flavanone |
| Kang – 2012 [62] | na | DMEM (glucose na) supplemented with 10% FBS and 1% penicillin/streptomycin | MTT assay | Immature C. sunki peel extract (CSE) (8, 40, 100, 200μg/mL) | DMSO | 24 h | Glycerol release  Western blot  qPCR | CSE (dose dependently):  ↑ glycerol release  ↑ pLKB1, pAMPK, and pACC protein levels  CSE (100 and 200μg/mL):  ↑ *Cpt1a* mRNA levels | Extract |
| Lasa – 2012 [42] | Cells seeded in 6-well plates | DMEM (glucose na) supplemented with 10% FBS and 1% penicillin/streptomycin | na | Trans-resveratrol (RSV), trans-resveratrol-3-O-glucuronide (3G), trans-resveratrol-4-O-glucuronide (4G) or trans-resveratrol-3-O-sulfate (3S) (1, 10 and 25μM) on day 12 after differentiation | 0.1% Ethanol | 24 h | Intracellular TG  qPCR | RSV (1-25μM) and 3G and 4G (10 and 25μM):  ↓ TG content  RSV (10μM):  ↑ ATGL, CPT-1b, PGC1α and SIRT-1 mRNA levels  4G (10μM):  ↑ HSL and SIRT-1 mRNA levels  3G (10μM):  ↓ FASN and SIRT-1 mRNA levels | Stilbene |
| Lasa – 2011 [43] | Cells seeded in 6-well plates | DMEM (glucose na) containing 10% FBS | na | Resveratrol (RS) (10 and 100µM) on day 12 after differentiation | 0.1 ethanol | 20 h | Intracellular TG  qPCR | RS (100µM):  ↓ TG content  ↑ ATGL mRNA levels | Stilbene |
| Mercader – 2011 [44] | Cells seeded in 6-well plates | DMEM (glucose na) supplemented with 10% NBC, 50 IU/ml penicillin, 50 μg/ml streptomycin and 2 mM l-glutamine | Lactate dehydrogenase activity | Resveratrol (RSV) (5, 10 and 20μM) on day 8 after differentiation | 0.1% DMSO | 24 and 72 h | Intracellular TG  Palmitate oxidation  qPCR  ELISA | RSV (5-20μM):  ↓ TG content  ↓ *Pparg*, *Glut4*, *Resistin*, *Rbp4* mRNA levels  ↓ resistin protein levels  RSV (20μM):  ↑ *Cpt1a* and  ↓ *Nrip1* mRNA levels  ↑ palmitate oxidation | Stilbene |
| Ju – 2011 [63] | na | DMEM (glucose na) supplemented with 10% BCS | MTT assay | Purple sweet potatoes (PSP) extract (1000, 2000 and 3000µg/mL) on day 8 after differentiation | Untreated adipocytes | 24 h | Oil Red O staining  Adipocyte size  qPCR  ELISA  Western blot | PSP extract (3000µg/mL):  ↓ lipid accumulation  PSP extract (1000-3000µg/mL):  ↓ cell size  ↓ leptin production  ↓ ACS, FAS, LPL and SREBP-1c mRNA levels and  ↑ HSL, pHSL, perilipin, ACO, CPT-1 and ACD protein levels | Extract |
| Jung – 2011 [64] | Cells seeded in 6 well plates at density of 1.25x10⁶ cells/well | DMEM high glucose supplemented with 10% FBS penicillin/streptomycin (100 U/mL/100 μg/mL | MTT assay | Citrus peel extract  (CPE) (50 and 500μg/mL) after differentiation | na | 13 d | Oil red O staining  TG content  GPDH activity  qPCR | CPE (50 and 500μg/mL):  ↓ lipid accumulation  ↓ TG content  ↓ GPDH activity  CPE (500μg/mL):  ↓ *Pparg*, *Srebp1*, *Cebpa*, Tf*ap2a,* L*pl*, *Fas*, H*sl*, *Plin1*, *Tnfa* mRNA levels | Extract |
| Zhang – 2010 [17] | Cells seeded in 96-well plates at density of 10⁴ cells/well | DMEM (glucose na) supplemented with 10% FBS | na | 7-O-methylaromadendrin (7-O-MA) (1 and 10μM) on day 12 after differentiation | Untreated cells | 1 h | Glucose uptake | 7-O-MA (1-10μM):  ↑ glucose uptake | flavonol |
| Lee – 2009 [30] | na | DMEM (glucose na) supplemented with 10% FBS, 2 mM glutamine, 100 U/mL penicillin and 100 μg/mL streptomycin | na | Epigallocatechin gallate (EGCG) (0.1 and 10μM) after differentiation | 0.01% DMSO | 24 h | Oil red O staining  Glycerol release  qPCR | EGCG (10μM):  ↓ lipid accumulation  ↑ glycerol release  ↑ *Hsl* mRNA levels | Flavan-3-ol |
| Rayalam – 2009 [20] | na | DMEM (glucose na)  supplemented with  10% BCS, penicillin/streptomycin (100 U/mL/100 μg/mL)  and 292 μg/mL of glutamine | MTS assay | Xanthohumol (XN) (25µM) | 0.2% DMSO | 12, 24 and 36 h | Oil red O staining  Caspase-3 and -7 assay  Lipolysis assay  ELISA  Western blot | XN (25μM):  ↑ glycerol release | Flavanone |
| Su – 2009 [33] | Cells seeded in 6 well plates | DMEM (glucose na) supplemented with 10% BCS and 1% antibiotic-antimycotic solution | na | Genistein (5μM) on day 8 after differentiation | DMSO | 48 h | qPCR  Western blot | Genistein (5μM):  ↓ Fasn and Scd1 mRNA levels  ↑ *Hsd11b1* mRNA and protein levels | Isoflavones |
| Pinent- 2005 [65] | na | DMEM (glucose na) supplemented with 10% FBS | na | Grape seed procyanidins extract (PE) (140 and 210mg/L) on day 10 after differentiation | na | 15 h  24, 48 and 72 h (Glycerol and NEFA release) | Oil red O staining  Glycerol and NEFA release  G3PDH activity  cAMP assay  qPCR | PE (140mg/L):  = lipid accumulation  ↑ Epinephrine-induced glycerol release  ↓ Glycerol and NEFA release  ↓ G3PDH activity  ↓ *Hsl* and *Pparg2* mRNA levels  PE at 210 mg/L:  ↑ AMPc levels | Extract |
| Mochizuki – 2004 [31] | na | DMEM (glucose na) supplemented with 10% CS, 100 U/ml penicillin and 10 µg/ml streptomycin | na | Epigallocatechin-3-gallate-L (EGCG-L) (0.01mg/mL), Epigallocatechin-3-gallate-H (EGCG-H) (0.02mg/mL), (+) catechin-L (CAT-L) (0.01mg/mL) and catechin-H (CAT-H) (0.02mg/mL) after differentiation | na | 4 h | Glycerol release | EGCG-L (0.01mg/mL) and EGCG-H (0.02mg/mL):  ↑ glycerol release | Flavan-3-ol |
| Ardévol – 2000 [32] | na | DMEM (glucose na)  Supplemented with 10% calf serum, 2mM glutamine,  100U/ml penicillin and 100/1.5 mg/ml streptomycin/fungizone. | Neutral red | Catechin, epicatechin and  grape seed procyanidin extracts (150µM) on day 8 after differentiation | Untreated cells | 0.5-24 h | Glycerol release  G3PD assay  Northern blot | Catechin and epicatechin (150µM):  = glycerol release  = G3PD activity  Grape seed procyanidin extracts(150µM):  ↑ glycerol release  ↓ G3PD activity  ↓ *Hsl* mRNA level | Flavan-3-ol  Extract |
| Harmon – 2000 [21] | Cell seeded in 96-well plates at a density of 10,000 cells/100 ml/well or in 24-well plates at a density of 50,000 cells/ml/  well or in 6-well plates at a density of 130,000 cells/2 ml/well | DMEM (glucose na) supplemented with 10% FBS, 1% penicillin-  streptomycin (10,000 U/ml penicillin and 10,000 mg/ml  streptomycin in 0.85% saline), and 1% 100 mM pyruvate | LDH assay | Genistein and naringenin (100µM) with or without epinephrine (0.1 and 1mM) | 0.1% DMSO | 24 h | Glycerol release | Genistein alone:  ↑ glycerol release  Genistein + epinephrine:  ↑↑ glycerol release | Isoflavones (genistein)  Flavanone (naringenin) |
